# Recurrent giant cell myocarditis following orthotopic heart transplant resulting in urgent redo orthotopic heart transplantation: a case report

**DOI:** 10.1093/ehjcr/ytad602

**Published:** 2023-11-27

**Authors:** Abdullah AlJohani, Joshua Solomon, Abdulaziz Joury, Renzo Cecere, Nadia Giannetti

**Affiliations:** Centre for Outcomes Research and Evaluation Research, Institute of the McGill University Health Centre, 1001 Bd Décarie, Montreal, Quebec H4A 3J1, Canada; Division of Cardiology, McGill University Health Centre, McGill University, 1001 Bd Décarie, Montreal, Quebec H4A 3J1, Canada; DREAM-CV Laboratory, McGill University Health Centre, McGill University, 1001 Bd Décarie, Montreal, Quebec H4A 3J1, Canada; National Guard Health Affairs, King Abdulaziz Cardiac Center, Riyadh, Saudi Arabia; Centre for Outcomes Research and Evaluation Research, Institute of the McGill University Health Centre, 1001 Bd Décarie, Montreal, Quebec H4A 3J1, Canada; Division of Cardiology, McGill University Health Centre, McGill University, 1001 Bd Décarie, Montreal, Quebec H4A 3J1, Canada; DREAM-CV Laboratory, McGill University Health Centre, McGill University, 1001 Bd Décarie, Montreal, Quebec H4A 3J1, Canada; Centre for Outcomes Research and Evaluation Research, Institute of the McGill University Health Centre, 1001 Bd Décarie, Montreal, Quebec H4A 3J1, Canada; Division of Cardiology, McGill University Health Centre, McGill University, 1001 Bd Décarie, Montreal, Quebec H4A 3J1, Canada; DREAM-CV Laboratory, McGill University Health Centre, McGill University, 1001 Bd Décarie, Montreal, Quebec H4A 3J1, Canada; King Salman Heart Center, King Fahad Medical City, Riyadh, Saudi Arabia; Centre for Outcomes Research and Evaluation Research, Institute of the McGill University Health Centre, 1001 Bd Décarie, Montreal, Quebec H4A 3J1, Canada; Division of Cardiology, McGill University Health Centre, McGill University, 1001 Bd Décarie, Montreal, Quebec H4A 3J1, Canada; DREAM-CV Laboratory, McGill University Health Centre, McGill University, 1001 Bd Décarie, Montreal, Quebec H4A 3J1, Canada; Department of Cardiovascular and Thoracic Surgery, Heart Failure and Transplant Center, McGill University Health Center, Montreal, Quebec, Canada; Centre for Outcomes Research and Evaluation Research, Institute of the McGill University Health Centre, 1001 Bd Décarie, Montreal, Quebec H4A 3J1, Canada; Division of Cardiology, McGill University Health Centre, McGill University, 1001 Bd Décarie, Montreal, Quebec H4A 3J1, Canada; DREAM-CV Laboratory, McGill University Health Centre, McGill University, 1001 Bd Décarie, Montreal, Quebec H4A 3J1, Canada

**Keywords:** Giant cell myocarditis, Heart transplant, Immunosuppression, Left ventricular assist device, Case report

## Abstract

**Background:**

Giant cell myocarditis (GCM) is a severe and rapidly progressing condition that can lead to end-stage heart failure. We present a case of a 51-year-old male with a history of orthotopic heart transplantation (OHTx) for GCM, who experienced recurrent GCM in the allograft, leading to progressive heart failure and the need for a second heart transplant.

**Case summary:**

A 51-year-old male with a history of OHTx for GCM presented with rapidly worsening heart failure symptoms. Despite initial stability, he deteriorated to cardiogenic shock and required intensive support. His clinical course was complicated by recurrent COVID-19 infections, worsened left ventricular ejection fraction, and withdrawal of guideline-directed medical therapy. Imaging showed extensive scar burden, and subsequent investigations ruled out coronary artery disease. With declining functional status and worsening cardiogenic shock, he was re-listed for OHTx and successfully underwent a second heart transplant.

**Discussion:**

Giant cell myocarditis poses challenges due to its aggressive nature. Early, aggressive immunosuppression and mechanical circulatory support are crucial. The recurrence rate of GCM post-OHTx is notable, often within the first year, and the optimal immunosuppressive regimen remains uncertain. In this case, GCM recurrence following OHTx led to continued deterioration despite treatment, necessitating a second heart transplant. This unique case emphasizes the complexity of managing recurrent GCM post-OHTx.

**Learning points**

- Giant cell myocarditis (GCM) is an acute, progressive form of inflammatory cardiomyopathy that typically affects middle-aged individuals with no prior medical history.
- The management of GCM consists of four cornerstones: immunosuppressive therapy, management of haemodynamic instability and ventricular arrhythmia, establishment of mechanical circulatory support, and consideration of orthotopic heart transplant (OHTx).
- Recurrence of GCM post-OHTx requires an intensification of immunosuppressive therapy, and re-initiation of advanced therapy may be considered.

## Introduction

Acute management of giant cell myocarditis (GCM) is challenging, given the acute haemodynamic instability (due to biventricular failure and/or unstable ventricular arrhythmias). Aggressive immunosuppressive therapy (combination of corticosteroids and calcineurin inhibitor) and rapid escalation of mechanical circulatory support (MCS) are cornerstones in the management of GCM.^1^ Orthotopic heart transplant (OHTx) has an evolving integral role in the management of refractory GCM.^1,2^ Here, we present a 51-year-old male with a prior OHTx due to GCM, who experienced an early recurrent GCM following OHTx leading to progressive heart failure and the need for a second heart transplant.

## Summary figure

**Figure:**
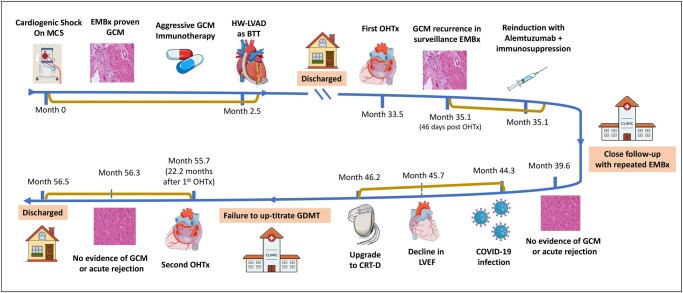


## Case presentation

**Figure 1:**
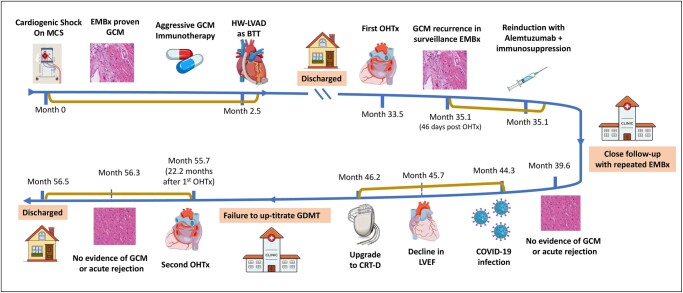
Timeline from the diagnosis of giant cell myocarditis to the second allograft orthotopic heart transplant. CRT-D, cardiac resynchronization therapy with defibrillator; GCM, giant cell myocarditis; GDMT, guideline-directed medical therapy; EMBx, endomyocardial biopsy; HW-LVAD, HeartWare left ventricular assist device; MCS, mechanical circulatory support; LVEF, left ventricular ejection fraction; OHTx, orthotropic heart transplant.

A 51-year-old male with a history of orthotopic heart transplantation for GCM presented to the emergency department with 2-week rapidly progressive symptoms of heart failure (i.e. worsening shortness of breath, orthopnoea, paroxysmal nocturnal dyspnoea, and weight gain). There was no history of cough, fever, or chest pain, and his review of the systems was grossly unremarkable. Physical examination revealed lethargic man and mild encephalopathy. He was cold and congested and in hypoperfusion state. He was initially haemodynamically stable with the Society for Cardiovascular Angiography and Interventions (SCAI) classification of cardiogenic shock C. Shortly after the presentation, he continued to deteriorate, despite support for inotropes, progressing to SCAI D cardiogenic shock, requiring rapid investigations and interventional planning (*Figure 1*).

**Figure 2:**
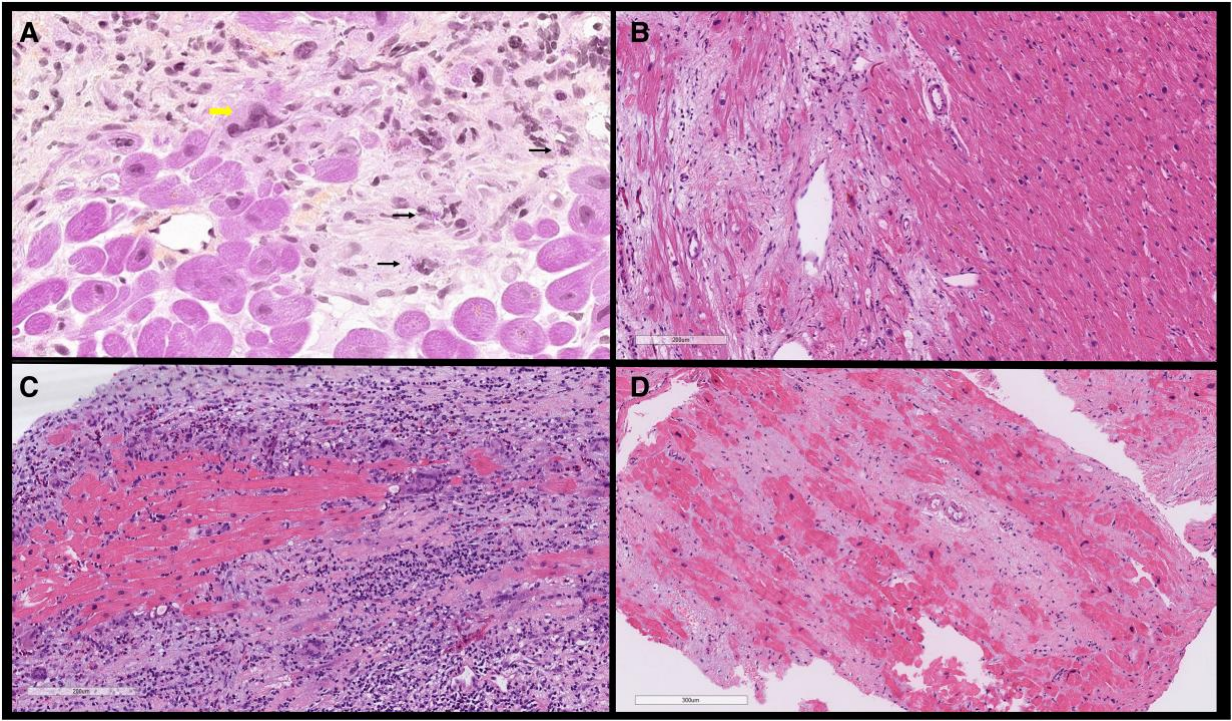
(*A*) Histology of the native heart showing inflammation and multinucleated giant cells. (*B*) Histopathology of the explanted heart showing the continuous presence of multinucleated giant cells. (*C*) Endomyocardial biopsy of the first heart transplant showing diffuse inflammation and scattered multinucleated giant cells, confirming the diagnosis of recurrence of giant cell myocarditis. (*D*) Biopsy of the first allograft heart after treatment for giant cell myocarditis, showing diffuse interstitial fibrosis.

Four years before this presentation, he was a healthy 46-year-old with no significant chronic medical illness and no autoimmune disease. He presented with the SCAI classification of cardiogenic shock D, which required temporary MCS in the form of venoarterial extracorporeal membrane oxygenation. His endomyocardial biopsy (EMBx) showed evidence of GCM (*Figure 2*). Following the diagnosis of GCM, he underwent aggressive immunotherapy with high-dose steroids, tacrolimus, and mycophenolic acid. He failed to be weaned down temporarily from MCS, and he eventually underwent a HeartWare left ventricular assist device (LVAD). Following LVAD, he could be discharged and was listed for OHTx.

Two years following LVAD, he underwent his first OHTx, where he was induced with basiliximab and was on a higher target immunosuppression with a combination of tacrolimus, mycophenolate mofetil, and prednisone (*Figure 1*). On his third surveillance EMBx (i.e. 46 days post-OHTx), his histopathology showed a recurrence of GCM in the allograft heart. He was asymptomatic, and his left ventricular ejection fraction (LVEF) was normal during and following this GCM recurrence. He was treated with alemtuzumab, rituximab, and high-dose steroids, which eradicated histopathologic evidence of residual GCM without affecting his LVEF. Within the first year of his first OHTx, he contracted coronavirus disease (COVID-19) and had a prolonged recovery. Sadly, during his recovery period from COVID-19, his LVEF continued to decline without EMBx evidence of GCM recurrence after intensifying his immune suppression.

During his recurrent COVID-19 infections, he was treated with sotrovimab, and he was introduced to guideline-directed medical therapy (GDMT) for heart failure with reduced ejection fraction (HFrEF). Subsequent imaging investigations showed worsening scar burden and a widening of the QRS complex where he eventually underwent cardiac resynchronization therapy with no benefit in the improvement of his LVEF. His clinical course continued to decline with the withdrawal of GDMT due to symptomatic hypotension, worsening kidney functions, and failure to thrive. The differential diagnosis for the current presentation was a recurrence of GCM in an allograft heart, COVID myocarditis, and progressive HFrEF.

**Figure 3:**
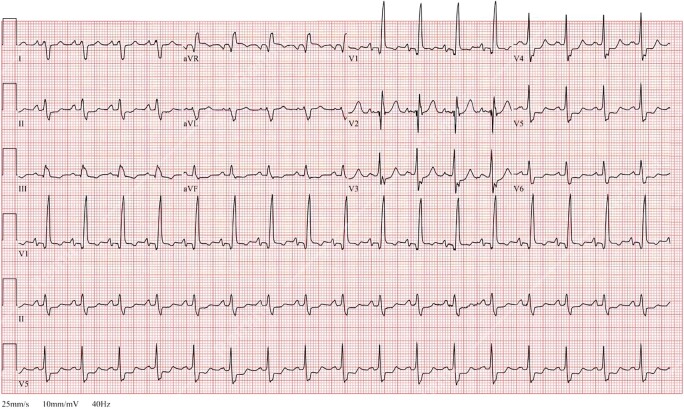
Electrocardiogram on presentation with progressive heart failure showing sinus rhythm with complete right bundle branch abnormality and left posterior fascicular block.

**Figure 4:**
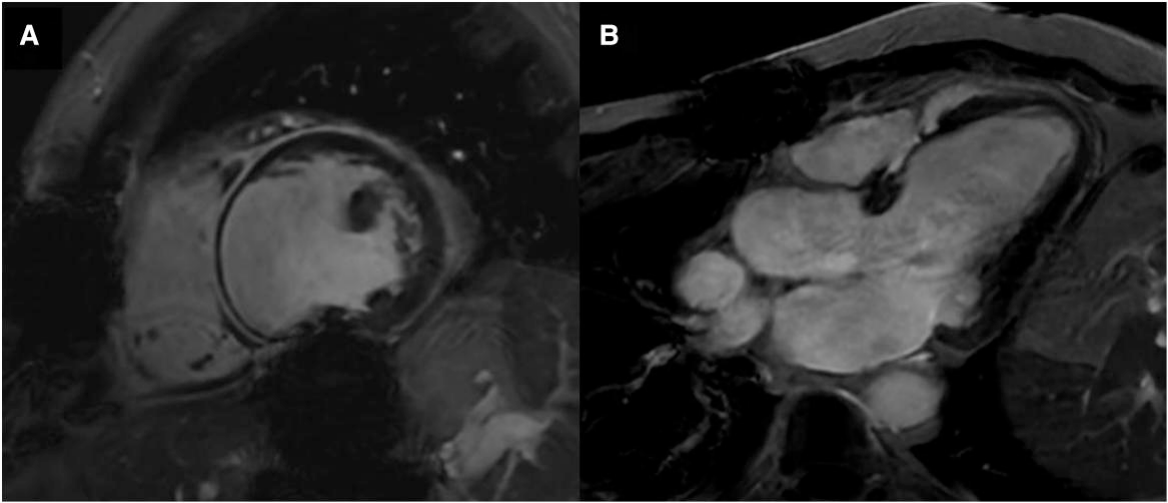
(*A* and *B*) Cardiac magnetic resonance imaging highlighting interval progression of an extensive predominantly subepicardial late gadolinium enhancement involving the basal to mid anterior, anteroseptal, and septal walls.

The initial laboratory investigations showed a high-sensitivity troponin I of 435 ng/L (normal range < 14 ng/L), NT pro-brain natriuretic peptide 16 218 pg/mL (normal range < 125 pg/mL), and lactic acid 2.4 mmol/L (normal range < 1.0 mmol/L). The transthoracic echocardiogram (TTE) showed a severely dilated and dysfunctional allograft with LVEF estimated at 15–20%. The electrocardiogram showed sinus tachycardia with a bifascicular block (*Figure 3*). Cardiac magnetic resonance imaging (cMRI) highlighted interval progression of an extensive predominantly subepicardial late gadolinium enhancement (LGE) involving the basal to mid anterior, anteroseptal, and septal walls (*Figure 4*). Given his history of GCM, he underwent another EMBx to exclude another recurrence of GCM in his transplanted allograft. His repeated EMBx showed no evidence of GCM recurrence; however, it showed an extensive myocardial scar (*Figure 2B* and *C*). To exclude coronary artery disease, he underwent coronary angiography, which showed no evidence of significant coronary artery disease as the culprit of his worsening HFrEF and cardiogenic shock (*Figure 4*).

This progressive decline in his functional status, escalating the requirement of diuretic doses and weaning off GDMT, alarms the need for re-consideration of advanced therapy for end-stage heart failure. In the first days following his admission, he showed multiple signs of worsening cardiogenic shock with SCAI classification of cardiogenic shock D. He required more inotrope support (i.e. milrinone 0.50 µg/kg/min). His laboratory investigation showed worsening NT pro-brain natriuretic peptide to 21 460 pg/mL and worsening lactic acidosis, and his repeated TTE showed worsening LVEF 10% and worsening of biventricular functions. He underwent right heart catheterization to determine his cardiac function and showed a cardiac index of 1.6 L/min/m^2^ and cardiac output of 3.1 L/min. He was urgently discussed in the multidisciplinary team, and the consensus was to proceed with re-listing for the second OHTx. A suitable donor was identified, and repeat heart transplantation was successfully performed within 72 h after enlistment. He was able to have uneventful post-operative course and was discharged home 6 days following his second OHTx. Multiple outpatient follow-up in heart transplantation clinic revealed stable clinical conditions and end-organ functions. His first and second EMBx following second OHTx revealed no evidence of rejection or recurrence of GCM.

## Discussion

Although the recurrence rate of GCM following OHTx has a wide range (14–47%)—commonly in the first year—however, the risk of GCM recurrence can be extended beyond 8 years following diagnosis.^2,3^ Despite these high rates of GCM following OHTx, the survival among GCM patients post-OHTx was similar to that of other aetiologies.^4^ Our patient had a recurrence of GCM in the second month post-OHTx, and he was managed with re-induction with alemtuzumab, rituximab, and high-dose steroids, which were successful in abate the GCM on histopathology (*Figure 2*). His LVEF continued to decline, which may raise a concern about the advanced stage of scarring mediated by recurrent GCM, as seen in his cMRI (*Figure 4*).

The combination of immunosuppressives to treat GCM was extrapolated from retrospective or small prospective studies; thus, the optimal combination of immunosuppression among GCM is yet to be discovered. Among patients with refractory GCM, alemtuzumab is considered as the appropriate therapy in refractory cases or in recurrent GCM.^5^ In our patient, we opted to use antithyroglobulin for induction therapy for his second OHTx, and we continued a combinational therapy of corticosteroids, tacrolimus, and mycophenolate mofetil. To the best of our knowledge, this is the first case reported of GCM recurrence in allograft OHTx that required a second OHTx. Our patient was discharged in a stable condition and assuring investigations. His first EMBx following the second OHTx showed no signs of acute rejection or GCM recurrence.

**Figure 5:**
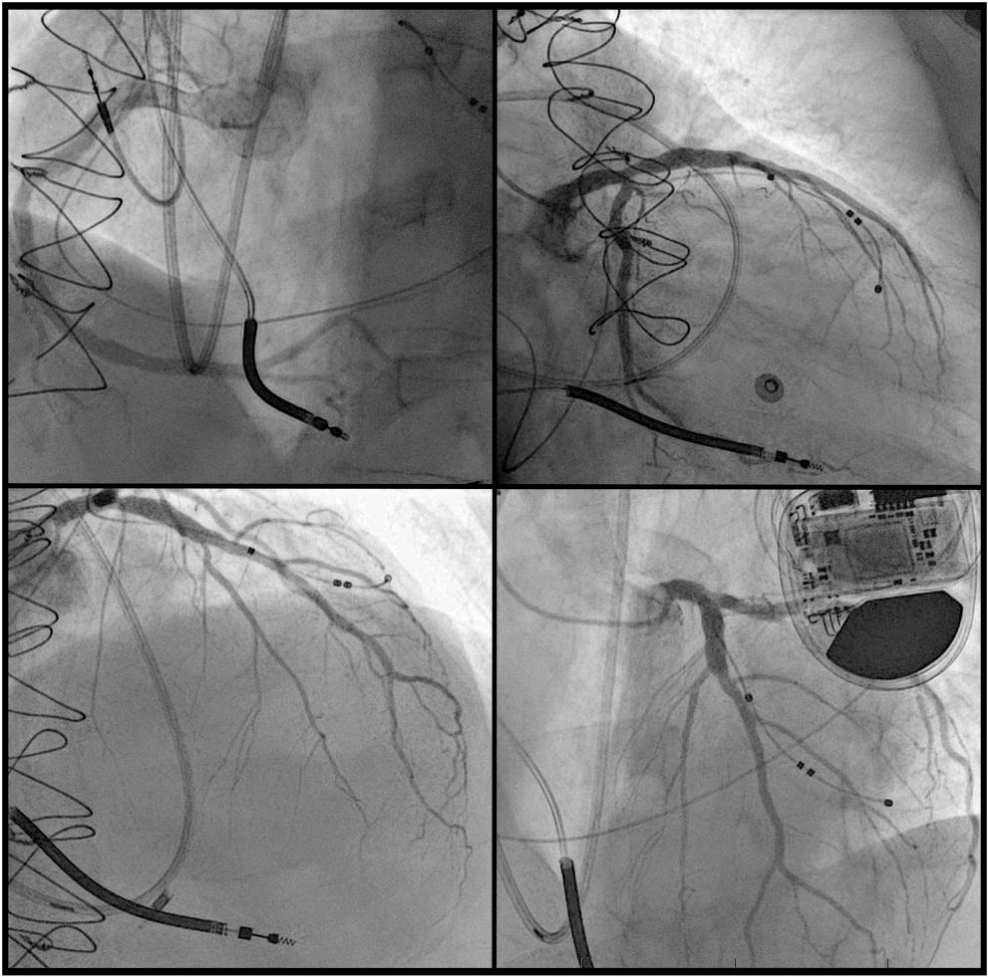
Coronary angiography of the first allograft heart showing non-obstructive coronary artery disease.

In conclusion, GCM is a disease that progresses rapidly and often results in fatality; thus, early and aggressive immunosuppression and MCS are essential. In this study, we present a case of a previously healthy middle-aged man who experienced GCM recurrence following OHTx, resulting in continued deterioration of his allograft heart and requiring a second OHTx (*Figure 5*).

## Data Availability

There are no new data associated with this article.

## Lead author biography

Dr Abdulaziz (Aziz) Joury is a distinguished medical professional with a strong background in cardiovascular medicine and clinical research. Holding an MBBS degree, he is currently a dedicated advanced heart failure and transplantation cardiologist at McGill University, Montreal, QC, Canada. Driven by a passion for advancing patient care, he has undergone comprehensive postgraduate clinical training, including an Internal Medicine and Cardiovascular Medicine Fellowship Program at Ochsner Medical Center, New Orleans, LA.

## Notes

**Consent:** We obtained an informed consent from the patient discussed in this case report. Patient’s consent was compliant with COPE guidelines.

**Funding:** None declared.
